# They Are Laughing at Me: Cerebral Mediation of Cognitive Biases in Social Anxiety

**DOI:** 10.1371/journal.pone.0099815

**Published:** 2014-06-11

**Authors:** Benjamin Kreifelts, Carolin Brück, Jan Ritter, Thomas Ethofer, Martin Domin, Martin Lotze, Heike Jacob, Sarah Schlipf, Dirk Wildgruber

**Affiliations:** 1 Department of Psychiatry and Psychotherapy, Eberhard Karls University of Tübingen, Tübingen, Germany; 2 Department of Biomedical Magnetic Resonance, Eberhard Karls University of Tübingen, Tübingen, Germany; 3 Department for Diagnostic Radiology and Neuroradiology, University of Greifswald, Greifswald, Germany; University of Leicester, United Kingdom

## Abstract

The fear of embarrassment and humiliation is the central element of social anxiety. This frequent condition is associated with cognitive biases indicating increased sensitivity to signals of social threat, which are assumed to play a causal role in the maintenance of social anxiety. Here, we employed laughter, a potent medium for the expression of acceptance and rejection, as an experimental stimulus in participants selected for varying degrees of social anxiety to identify cerebral mediators of cognitive biases in social anxiety using functional magnetic resonance imaging in combination with mediation analysis. We directly demonstrated that cerebral activation patterns within the dorsal attention network including the left dorsolateral and dorsomedial prefrontal cortex mediate the influence of social anxiety on laughter perception. This mediation proved to be specific for social anxiety after correction for measures of general state and trait anxiety and occurred most prominently under bimodal audiovisual laughter presentation when compared with monomodal auditory or visual laughter cues. Considering the possibility to modulate cognitive biases and cerebral activity by neuropsychological trainings, non-invasive electrophysiological stimulation and psychotherapy, this study represents a starting point for a whole line of translational research projects and identifies promising targets for electrophysiological interventions aiming to alleviate cognitive biases and symptom severity in social anxiety.

## Introduction

Social anxiety (SA), in its core, is the fear of embarrassment and humiliation in social situations. It is found in a spectrum of varying severity in the population [1]. The pathological end of this spectrum is called social anxiety disorder (SAD) and represents one of the most prevalent psychiatric conditions [1], [2]. It causes a severe loss of quality of life [3] and high economical costs [4]. Moreover, SA is associated with biased cognition. An attention bias with faster responses to socially threatening cues [5], [6] and a negative interpretation bias for facial [7] and vocal [8] expressions are among the most well known cognitive biases in SA. Critically, these biases are thought to play a causal role in the maintenance of clinical anxiety [9], [10]. Despite this assumed pivotal role of cognitive biases in SA, their neural underpinnings are still not well understood.

While neuroimaging studies in SA revealed predominantly increased cerebral activation to facial [11]–[15] and vocal [16] expressions signaling social threat in the limbic system [12]–[15], as well as increased activation of secondary visual cortices [12], [15], the mediofrontal cortex [11], [12], [14] and the orbitofrontal cortex for threatening voices [16], still none of the associated cognitive biases have been explicitly investigated in neuroimaging studies on SA based on their behavioral correlates. First evidence from studies in healthy participants [17] as well as patients with generalized anxiety disorder [18]–[20], posttraumatic stress disorder [21], or panic disorder [22] suggests an involvement of dorsolateral [17], [19]–[21], dorsomedial [21], and ventrolateral [18], [21], [22] aspects of the prefrontal cortex in the processing of the attention bias towards threat-related stimuli. Of these, solely the study by Browning et al. (2010) directly focused on the cerebral correlates of the attention bias itself and demonstrated decreased prefrontal activation to attended threat-signaling faces after induction of an attention bias towards such stimuli.

It was, thus, the aim of the present functional magnetic resonance imaging (fMRI) study to reveal the cerebral mediators of threat-related cognitive biases in SA using audiovisual recordings of laughter as a novel symptom-provoking tool.

Laughter represents a primordial social communication signal which is already present in non-human primates [23]. It offers an excellent opportunity to investigate cognitive biases in SA as it evolved into different laughter types in humans which can serve group bonding [24] (e.g. joyful laughter with a positive valence) but also social segregation [25] (e.g. taunting laughter with a negative valence). In contrast to these evolutionary younger laughter types, tickling laughter serves the enforcement of play behavior [26] and is confined to the context of bodily contact. It has been demonstrated that different laughter types are distinguishable based on the vocal signal [27] and cerebral activation patterns [28], [29].

Taken together, in the context of SA where central fears pertain to humiliation, criticism and rejection, and where laughter has been shown to exhibit phobogenic properties [30], laughter represents an ideal stimulus to evoke typical behavioral correlates of SA.

Twenty-four participants with a broad spectrum of SA severity ranging from very low to clinical social anxiety (i.e., SAD) took part in the present study. They were confronted with different laughter types (i.e., joyful, taunting and tickling laughter) and asked to rate to which degree the laughter was socially inclusive or exclusive on a four-point scale.

Based on previous findings obtained for facial expressions [5], [6], we hypothesized a linear relationship between SA and faster response times to socially exclusive taunting laughter than to socially inclusive joyful laughter. Moreover, we expected that, joyful and taunting laughter would be rated as more socially rejecting with increasing severity of SA, while no such effect would occur for the reflex-like tickling laughter due to its lack of social communicative functions outside the context of direct bodily contact during play behavior.

To evaluate potential effects of the sensory modality of cue presentation, the experimental design included auditory, visual, and audiovisual trials. Laughter ratings and response times were recorded as behavioral outcome parameters. The Liebowitz Social Anxiety Scale (LSAS) was used as measure of SA while the State-Trait-Anxiety-Inventory (STAI) was employed to capture general anxiety as a control variable.

The main goal of the present study was to establish the associations between SA (as obtained by the Liebowitz Social Anxiety Scale), behavioral correlates of cognitive biases during laughter perception and cerebral activation using mediation analysis. This technique which has been recently introduced in imaging neuroscience [31] allows the clarification of causal inferences on the relationship of behavioral dispositions, brain activity and behavior.

To this end, we first investigated the influence of SA on laughter ratings and response times. Then, we identified cerebral structures in which activation alterations reflected changes in these behavioral measures. Based on the study by Browning et al. (2010) on the cerebral correlates of the attention bias towards threatening faces and the neuroimaging studies evidencing bias-congruent decreased activation of the prefrontal cortex (PFC) cues in patients with different anxiety disorders [18]–[22] described above, we hypothesized a positive correlation between faster responses and decreased activation to taunting as compared to joyful laughter in the PFC.

Subsequently, it was tested if regions with a significant relationship between cerebral activation and SA-associated cognitive biases also exhibited a significant linear association of their activation with the severity of SA.

Finally, we performed a mediation analysis to clarify the interdependency of the severity of SA, cognitive biases, and cerebral activation within these brain areas. Validation analyses included a bootstrapping approach controlling for effects of general anxiety as well as an investigation of effects of cue presentation modality on the relationships between cognitive biases as well as SA and cerebral activation patterns to detect potential modality-dependent dissociations between behavior, cerebral activation patterns and SA.

## Materials and Methods

### Ethics Statement

This study was reviewed and approved by the University of Tübingen ethical review board before the study began. The study was performed in accordance with the Code of Ethics of the World Medical Association (Declaration of Helsinki), and all participants gave written informed consent according to the guidelines of the University of Tübingen ethical review board prior to their inclusion in the study. The individual in this manuscript has given written informed consent (as outlined in the PLOS consent form) to publish her picture which is a still image taken from the employed stimulus material.

### Participants

Based on the observation that the severity of SA and its transition into SAD represent a continuum in the population rather than categories of non-clinical and clinical anxiety [32], [33], a single group of participants with a wide variation of SA severity ranging from minimal SA to severe SAD was included in the present study.

Twenty-four volunteers (11 male, 13 female, mean age 25.3, SD 3.4 years) were recruited for the study through advertisement bulletins. All participants were native German speakers and were right-handed, as assessed with the Edinburgh Inventory [34]. None of the participants had a history of neurological or psychiatric illness, substance abuse or impaired hearing, or was on any medication. Vision was normal or corrected to normal. Before inclusion in the study all participants were screened for psychiatric disorders by a psychiatrist (S.S.) using the Structured Clinical Interview for DSM-IV [35] screening questions. In cases where at least one screening questions was answered in the affirmative, the full interview was administered. LSAS [36], German self-report version, was used to measure severity of SA. The six participants with the highest LSAS self-ratings fulfilled the clinical criteria of SAD. General anxiety was assessed using the State-Trait-Anxiety-Inventory (STAI, German version [37]. The “Mehrfachwahl-Wortschatz-Intelligenz-Test” (MWT-B, [38]) was applied to measure premorbid intelligence. The socio-demographic and psychometric study population data are given in Table 1.

**Table 1:** Socio-demographic and psychometric data.

	mean (SD)
age (years)	25.3 (3.4)
years of education	15.6 (1.8)
MWT-B	30.8 (3.3)
LSAS	32.2 (34.1)
STAI state (X1)	36.1 (7.4)
STAI trait (X2)	44.6 (2.2)

MWT-B = “Mehrfachwahl-Wortschatz-Intelligenz-Test”, a short test of premorbid intelligence; LSAS = Liebowitz Social Anxiety Scale; STAI = State Trait Anxiety Inventory.

### Stimuli and Task

Sixty short video portrayals (1.5 s) of laughter (i.e. laughing faces) were used as stimulus material. The stimuli were part of a larger corpus of video portrayals (187 sequences) including three types of laughter (joyful/friendly [JOY], tickling [TIC] and taunting/unfriendly [TAU] laughter). The stimuli were produced by eight professional actors using a script-based auto-induction technique. The video footage was post-processed to ensure a comparable video (Adobe Premiere, Pro CS3, Adobe Systems, Inc., San Jose, CA, USA) and sound (PRAAT, 5.1.07) quality across recordings. Audio recordings were normalized for mean acoustic energy, and videos were post-processed so that the facial symmetry axis was vertical, and head size was comparable across videos.

The post-processed stimuli were evaluated in two behavioral prestudies by healthy participants with regard to the categorical recognizability of the expressed laughter type (n = 14, mean age 24.4, SD 2.4 years) and authenticity (n = 14, mean age 24.3, SD 3.5 years). Only stimuli with recognition rates exceeding chance level and with at least average authenticity ratings (i.e., >3.0 on a 9-point-scale; mean authenticity 5.1, SD 1.4) were selected for the fMRI study. The stimulus set was balanced for laughter type and gender of the speakers (JOY = 8 m/10 f, TIC = 9 m/11 f, TAU = 10 m/12 f), as well as the recognition rates of the three laughter types (unbiased hit rates [39]: JOY = 0.52, TIC = 0.54, TAU = 0.53; paired t-tests: t(13) ≤0.5, P≥0.6).

During the fMRI experiment all sixty stimuli were presented under three different conditions (auditory [A], visual [V] and audiovisual [AV]; total number of stimuli = 180) within an event-related design. The experiment consisted of three runs with 60 stimuli each. The runs were balanced for laughter type and presentation modality (A, V, AV). The stimulus sequence within each run was randomized, and the sequence of the runs was balanced across the participants.

**Figure 1:**
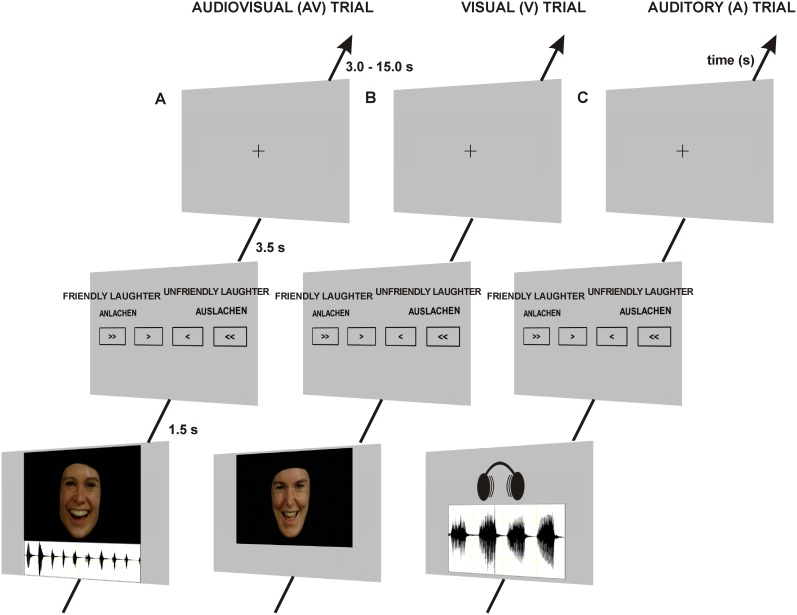
Trial design. The figure shows three exemplary experimental trials (a–c). (a) illustrates a trial with audiovisual (AV) laughter presentation while (b) and (c) show visual (V) and auditory (A) trials, respectively. The participants’ task was to evaluate on a four-point scale if and how clearly the laughter expressed a friendly (German: “Anlachen”) or an unfriendly (German: “Auslachen”) social intention. The English expressions “FRIENDLY LAUGHTER” and “UNFRIENDLY LAUGHTER” above the German expressions “ANLACHEN” and “AUSLACHEN” are not part of the original scale shown in the experiment but were inserted to enhance the comprehensibility of the figure. Time specifications on the time axis indicate the durations of stimulus presentation, additional response window and inter-trial-interval.

Each trial (see Figure 1) began with the presentation of a laughter sequence. It was the participants’ task to decide if the laughter sequence bore a friendly or an unfriendly social intention. They were instructed to imagine that the laughter was directed at them by the laughing person they saw and/or heard. Furthermore, the participants were instructed not to laugh during the experiment. To prevent a central tendency, responses were given on a 4-point scale (see Figure 1) with the symbols “>>”, “>”, “<”, “<<” and the German words “Anlachen” (friendly laughter) and “Auslachen” (unfriendly laughter) at opposite ends of the scale. “>>” indicated the decision that the laughter clearly belonged to the laughter category the name of which the open sides of the symbols were pointing to. “>” indicated that the participants judged the laughter sequence as rather belonging to the respective category but were not sure. To avoid effects attributable to the arrangement of response alternatives, the response scale was flipped horizontally for half of the participants. The participants were instructed to give their response as quickly as possible by pressing one of four buttons on a fiber optic response system (LUMItouch, Photon Control, Inc., Burnaby, BC, Canada) placed under their right hand. Responses were required within a time frame of 5 s following stimulus onset. “Presentation” (Neurobehavioral Systems, Inc., Albany, CA, USA) was used as software for stimulus delivery. Video sequences employed in the present study were back-projected onto a screen placed in the magnet bore behind the participant’s head and viewed by the participant through a mirror system mounted onto the head coil. Laughter sounds were presented via MR compatible headphones (MR confon GmbH, Magdeburg, Germany). The onsets of the stimuli were jittered relative to the scan onsets in steps of 500 ms (1/4 of the repetition time (TR)  = 2000 ms) to reduce effects of stimulus expectancy. Null events with durations of 10 s were randomly inserted in the trial sequence with the frequency of one null event per 10 trials.

### Image Acquisition

A Siemens MAGNETOM Verio 3-Tesla whole-body MRI scanner (Siemens AG, Erlangen, Germany) was used for the MR measurements. For functional imaging an echo-planar imaging (EPI) sequence (TR = 2000 ms, echo time [TE] = 30 ms, flip angle = 90°, field of view = 192×192 mm^2^, 64×64 matrix, 34 slices, 3 mm slice thickness, 1 mm gap, orientated along the anterior commissure-posterior commisure plane) was employed. For the anatomical reference high-resolution T1-weighted images a magnetization-prepared rapid acquisition gradient echo sequence (TR = 1900 ms, TE = 2.52 ms, 176 slices, 1 mm thickness) was used. For the off-line correction of EPI image distortions, a static fieldmap (34 slices, TR = 488 ms, TE1 = 4.92 ms, TE (2)  = 7.38 ms, α = 60°) was acquired.

### Analysis of Study Population Data and Behavioral Data

IBM SPSS Statistics Version 19 (IBM Corp., Armonk, NY, USA) was used for statistical analyses. Analogue behavioral ratings were transformed to numerical values between 1 (clearly “Auslachen”, i.e. unfriendly laughter) and 4 (clearly “Anlachen”, i.e. friendly laughter). In order to avoid biases in the response time data due to extreme outliers based on inattention, all responses above two standard deviations from the individual mean response time were excluded from further analysis of the response time data. A normal distribution of behavioral data and population parameters was ascertained using the Kolmogorov-Smirnov test (with the exception of gender). LSAS scores were tested for a linear association with the other population parameters (age, gender, years of education, MWT-B and STAI-X1/X2) using bivariate correlation analyses. Based on the specific hypotheses of the present study, linear regression analyses investigating the following linear associations were performed:

- LSAS and mean overall laughter ratings as well as the laughter ratings for the separate laughter types and
- LSAS and mean response time differences between JOY and TAU.

Then it was tested if the linear relationship between SA and mean ratings differed between the three laughter types using the approach described by Meng et al. [40] and if the linear association between SA and laughter ratings was significant for the separate laughter types. The approach of Meng et al. [40] was also used to investigate if the strengths of any observed linear relationships between SA and behavioral measures were dependent on the modality of cue presentation (A, V, AV) and if this association was significant for each and every cue presentation modality. Here, the results were Bonferoni-corrected due to the lack of a priori hypotheses. These analyses were supplemented by two exploratory analyses of variance (ANOVA) with laughter ratings and response times as dependent variables and cue modality (A, V, AV) and laughter type (JOY, TIC, TAU) as within-subject factors and LSAS values as a covariate. These analyses were used to detect further potential associations between LSAS scores and the experimental factors (cue modality, laughter type) outside the scope of our a priori hypotheses and were therefore solely focused on potential interaction between the experimental factors and SA. One-tailed P values were used for tests with a directional hypothesis and two-tailed P values otherwise.

### Image Analysis

The SPM8 software package (Wellcome Department of Imaging Neuroscience, London, UK; http://www.fil.ion.ucl.ac.uk/spm) was used to analyze the functional images. The first five EPI images from each run were discarded to exclude measurements preceding T1 equilibrium. The images were realigned to the first volume of the time series, unwarped using a static field map, normalized to the Montreal Neurological Institute (MNI) space (resampled voxel size: 3×3×3 mm^3^) and spatially smoothed with an isotropic Gaussian kernel (8 mm full width at half maximum). Statistical analysis relied on a general linear model [41]. A separate regressor was defined for each event using a stick function convolved with the hemodynamic response function. Events were time-locked to stimulus onset. To remove low-frequency components, a high-pass filter with a cutoff frequency of 1/128 Hz was employed. The error term was modeled as a first-order autoregressive process with a coefficient of 0.2 and a white noise component to account for serial autocorrelations.

In accordance with this study’s aim, the analytic strategy proceeded in three steps:

1. The cerebral correlates of SA-associated behavioral responses during laughter perception were investigated. To this end, individual (first level) hemodynamic contrasts were calculated for those comparisons yielding a significant linear relationship between the equivalent contrast at the behavioral level and SA. These individual contrasts and the respective individual behavioral measurement (laughter ratings, response times) mean values were correlated at the group level. Statistical inference was based on second-level random effects analyses. Activations are reported at a height threshold of P<0.001, uncorrected, and an extent threshold of k>10 voxels. Significance was assessed at the cluster level with an extent threshold of P<0.05 (corresponding to a minimal cluster size of 60 voxels), FWE corrected for multiple comparisons across the whole brain.
2. The mean contrast estimates were extracted from clusters with a significant linear relationship between SA-associated behavioral measures during laughter perception and the respective cerebral hemodynamic activation. Then, it was tested whether cerebral responses in these clusters are also correlated with SA, as obtained by LSAS scores. Additionally, it was investigated if observed associations between the cerebral activation contrasts, on the one hand, and the behavioral correlates of cognitive biases during laughter perception as well as SA depended on the cue presentation modality. The approach suggested by Weaver and Wuensch [42] for comparing two non-independent correlations with no variables in common was used for testing the potential impact of cue modality on the linear relationship between cerebral activation and cognitive biases while the Meng test [40] was used to investigate cue modality effects on the relationship between SA and cerebral activation contrasts. All tests where Bonferoni-corrected for the number of performed comparisons (3).
3. In clusters where SA, behavioral responses and cerebral responses were all significantly intercorrelated, the Sobel test [43] was employed to test a potential mediation of the association of SA and behavioral responses through the respective cerebral responses during laughter perception. SA was defined as the independent variable *X* while the associated behavioral responses during laughter perception were defined as the dependent variable *Y*. The mean contrast estimates from the cluster of interest were entered as the potential mediator *M*. The total effect of *X* on *Y* was termed *c*, while *c’* was the direct effect of *X* on *Y*. The effects of *X* on the potential mediator *M* was termed a, and that of the potential mediator on *Y* was termed *b*. Thus, the indirect effect of *X* on *Y* through the potential mediator *M* was calculated as *ab*. This analysis was validated by a bootstrapping approach [44] (5000 resamples) which does not rely on the assumption of a normal distribution of the data. Here, results are given as 95% confidence intervals (CI) of the estimated indirect, i.e. mediated, effect. To investigate if any observed mediation effects were specific for SA, a post-hoc mediation analyses were performed where general state (STAI-X1) and trait (STAI-X2) anxiety were included as covariate of no interest. To determine if the results of this analysis were valid for each and every cue presentation modality, it was repeated for each separate cue modality and results were Bonferoni-corrected (3).

## Results

### Population Parameters

SA (as determined by LSAS scores) exhibited a significant positive correlation with general state (STAI-X1; r = 0.62, P = 0.001) and trait (STAI-X2; r = 0.42, P = 0.04) anxiety but not with any of the other population parameters (all r≤0.38; P>0.05).

### Behavioral Responses

#### Laughter ratings

SA was positively correlated with an increasing tendency to rate laughter as unfriendly (β = −0.006, t(22)  = 4.5, P<0.001, mean overall laughter rating ± SEM: 2.38±0.06). This linear relationship was significant for all three laughter types (all β≤−0.006, all t(22) ≥1.7, all P<0.05, mean ratings ± SEM: JOY: 2.00±0.05, TIC: 2.25±0.12, TAU: 2.90±0.06) and cue modalities (all β≤−0.006, t(22) ≥3.0, P≤0.018, mean ratings ± SEM: A: 2.30±0.07, V: 2.59±0.07, AV: 2.26±0.07), and there were no significant differences in this association between the different cue presentation modalities (all Z≤1.3, all P>0.05). However, the negative linear relationship between laughter ratings and SA was stronger for both TAU and JOY as compared to TIC (Z≥2.1, P≤0.016). The additional exploratory ANOVA did not reveal any significant interactions between the experimental factors and LSAS scores with regard to laughter ratings (all F≤1.2, all P>0.05).

#### Response times

**Figure 2:**
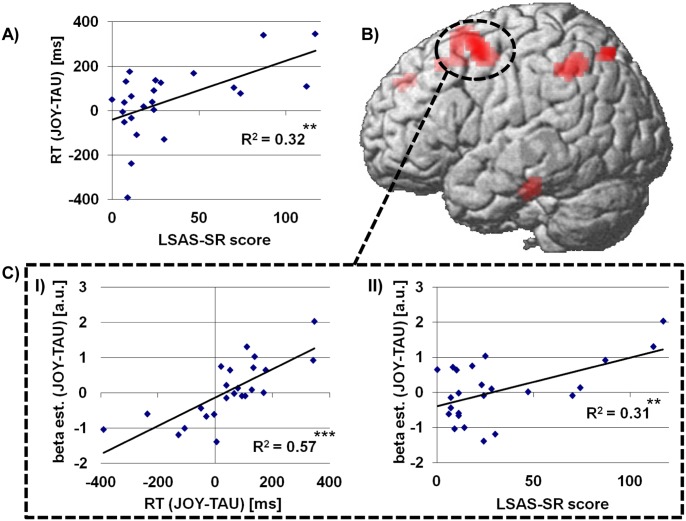
Behavioral and cerebral correlates of the negative attention bias during laughter perception in individuals with varying severity of social anxiety. a) Linear relationship between LSAS-SR scores and faster response times to taunting (TAU) than to joyful (JOY) laughter, i.e. negative attention bias. b) Cerebral correlates of the negative attention bias in the left DLPFC (circled). Results are shown at a threshold of p<0.001, uncorrected at voxel-level, and a cluster extent of k>10 voxels; results in the DLPFC are significant with p<0.05, FWE-corrected for multiple comparisons across the whole brain at cluster level. c) ROI analysis of extracted mean parameter estimates in the left DLPFC (I) illustrates the linear association of the negative attention bias and cerebral activation during laughter perception and (II) demonstrates an additional positive linear association of differential responses to TAU and JOY in this region and LSAS-SR scores. Asterisks mark levels of significance: **p<0.01, ***p<0.001.

A positive linear relationship between increasing SA and mean response time differences of JOY and TAU was observed (β = 2.7, t(22)  = 3.2, P = 0.004, see Figure 2-a; mean response time difference ± SEM: 45 ms±33 ms). No such association was observed for mean overall response times (β = 2.5; t(22)  = 1.0, P>0.05, mean overall response time ± SEM: 2354 ms±82 ms), mean response times for the different laughter types (all β≤3.8, all t(22) ≤1.7, all P>0.05, mean response times ± SEM: JOY: 2353 ms±80 ms, TIC: 2398 ms±93 ms, TAU: 2309 ms±79 ms), cue modalities (all β≤3.0, all t(22) ≤1.3, all P, mean response times ± SEM: A: 2449 ms±86 ms, V: 2312 ms±83 ms, AV: 2300 ms±80 ms) or other mean response time differences (i.e. between TIC and TAU or JOY or between A, V and AV (all β≤1.6, all t(22) ≤1.4, all P>0.05). Also, the exploratory ANOVA with response times as dependent variable did not reveal any significant interactions between the experimental factors and LSAS scores (all F≤3.2, all P>0.05).

### Cerebral Responses

#### Relationship of SA-associated behavioral responses and cerebral responses

For the SA-associated response time differences between JOY and TAU the whole-brain analysis revealed a significant positive linear relationship with the cerebral responses (contrast of interest: JOY – TAU) in the left dorsolateral prefrontal cortex (DLPFC; see Table 2 and Figure 2-b and 2-c-I).

**Table 2:** Brain areas where the attentional bias during laughter perception (response times JOY – TAU) was positively correlated with stronger responses to JOY than to TAU.

	x	y	z	Z-score (peak voxel)	Cluster size (voxel)
L cerebellum/L parahippocampal gyrus/L fusiform gyrus	–18	–27	–24	4.58	39
R superior and middle frontal gyri	24	45	36	4.15	32
L superior and middle frontal gyri/L precentral gyrus	–21	3	54	4.14	86*
R superior parietal gyrus/R angular gyrus	30	–69	51	3.96	43
R and L supplemental motor area/superior frontal gyrus pars medialis	9	15	63	3.84	53
L inferior parietal gyrus	–36	–51	45	3.86	32
L superior parietal gyrus	–24	–72	54	3.43	19

Activations thresholded at p<0.001, uncorrected with a cluster size k≥10 voxels. Coordinates refer to the MNI system. Only those clusters whose size is marked with a “*” can be considered significant at p<0.05, FWE corrected for multiple comparisons across the whole brain at the cluster level which corresponds to a cluster size of k≥60 voxels. Clusters with a size k<60 voxels are shown solely for the purpose of completeness. Voxel size 3×3×3 mm^3^.

The investigation of a linear relationship of overall mean laughter ratings with the respective cerebral responses to laughter did not yield any significant results (see Table S1).

#### Relationship of SA and cerebral responses

The regression analysis of the cerebral activation patterns of the DLPFC on the LSAS scores indicated that differential cerebral responses to JOY and TAU were positively associated with the severity of SA across participants (DLPFC: β = 0.14, t(22)  = 3.2, P = 0.005, see Figure 2-c-II).

#### Mediation analyses

**Figure 3:**
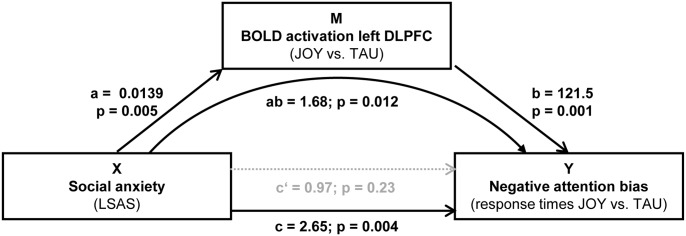
Mediation of the impact of social anxiety on the negative attention bias during laughter perception through cerebral activation patterns of the prefrontal cortex. X = independent variable (i.e., LSAS-SR scores), Y = dependent variable (i.e., response time differences JOY – TAU), M = mediator variable (i.e., differential cerebral activation JOY – TAU in the left DLPFC); a = effect of X on M; b = effect of M on Y partialling out X; c = total effect of X on Y; c’ = direct effect of X on Y; ab = indirect effect of X on Y through M. p value of ab refers to the Sobel test. Continuous arrows indicate significant effect while non-significant effects are symbolized by dashed arrows. Significance of the results remains unaltered when statistically controlling for the influence of general anxiety (STAI).

The conducted mediation analysis demonstrated that cerebral activation patterns within the left DLPFC mediated the relationship between SA and the negative attention bias during laughter perception even when general trait (STAI-X2) or state (STAI-X1) anxiety were included in the model as covariates of no interest (see Figure 3 and Table 3).

**Table 3:** The impact of social anxiety (X) on the attentional bias (Y) during laughter perception (response times JOY – TAU) was mediated through cerebral activation (M) patterns in left DLPFC.

control variable			X → Y		X → M	M → Y
		total effect	direct effect	indirect effect		
		c	c’	ab	a	b
	beta	**2.652**	0.968	**1.684**	**0.014**	**121.5**
none	Z	**2.9**	1.2	**2.5**	**2.8**	**3.3**
	P value	**0.004**	0.227	**0.012**	**0.005**	**0.001**
	95% CI			**3.768–0.671**		
	beta	**2.257**	0.182	**2.439**	**0.018**	**136.2**
STAI-X1	Z	**2.0**	0.2	**-**	**2.7**	**3.7**
	P value	**0.046**	0.849	**-**	**0.004**	**<0.001**
	95% CI			**4.126–0.846**		
	beta	**2.123**	0.892	**1.233**	**0.011**	**116.3**
STAI-X2	Z	**2.2**	1.1		**2.1**	**3.0**
	P value	**0.027**	0.284		**0.034**	**0.003**
	95% CI			**3.294–0.298**		

Mediation analysis: X = independent variable, Y = dependent variable, M = mediator, a = influence of X on M, b = influence of M on Y controlling for X, c = total effect of X on Y, c’ = direct effect of X on Y, ab = indirect effect of X on Y through M. Z values for the ab path pertain to the Sobel test while 95% CI pertain to the bootstrapping approach (for details see Methods). Values displayed in bold type refer to significant effects.

#### Effects of cue modality on the relationships between cerebral and behavioral responses as well as SA

The decomposition of the linear associations between SA-related behavioral responses and cerebral responses across cue presentation modalities revealed a tendency towards a stronger association between the negative attention bias and the respective cerebral activation patterns under AV than under V stimulation (Z = 2.2, P = 0.08) while the results of the remaining intermodal comparisons were non-significant (all Z≤1.1, all P>0.05). Moreover, the association between the negative attention bias and cerebral activation under the V condition was not significant (β = 0.002, t(22)  = 1.5, P>0.05).

For the correlation between the activation patterns (JOY–TAU) in the left DLPFC and LSAS no modality-dependent differences were observed (all Z≤1.3, all P>0.05).

Confirmatory mediation analyses for the separate modalities of cue presentation, however, yielded significant results only for AV (see Table S2).

## Discussion

This is, to our knowledge, the first neuroimaging study employing laughter to evaluate cognitive biases in individuals with varying degrees of SA. Here, we combined event-related fMRI with mediation analysis to reveal specific explanatory cerebral activation patterns for the influence of SA on the negative attention bias during laughter perception.

Our study identified the left DLPFC as key structure linking SA and the negative attention bias. Keeping in mind that faster responses to socially exclusive laughter were correlated with weaker cerebral responses to this laughter type, these results fit well with an earlier report of decreased activation of the lateral prefrontal cortex and faster responses to aversive stimuli in healthy individuals trained to attend to such stimuli [17].

### The Negative Attention Bias and the Dorsal Attention System

The left DLPFC is part of the dorsal frontoparietal attention network which is involved in goal-directed stimulus processing and response selection [45]. Both, goal-directed stimulus processing [46] and the function of the dorsal attention network [47] have been found to be impaired in highly anxious individuals. The hypothesis that the observed behavioral alterations in individuals with pronounced SA are related to the dorsal attention system is further supported by additional, even though not significant in the whole-brain analysis, linear associations of the attention bias and cerebral activation in areas adjacent to the bilateral intraparietal sulci (see Table 2) also part of the dorsal attention system. A question arising from the present findings is how the association of faster response times to socially rejecting laughter and decreased cerebral activation can be related to the function of the underlying network. Presumably, the observed reduced cerebral activation to threatening stimuli in high SA individuals reflects less effortful processing of such stimuli due to increased expectation of threatening social signals in high SA individuals. Furthermore, bearing in mind that the contrast of interest in the present study also contained positive social stimuli, a recently demonstrated tendency in SA to allocate attention away from such stimuli [48] could offer an additional or alternative explanation of the observed behavioral and neuronal effects.

### Translational Perspectives

As the negative attention bias in SA is thought to play a causal role in the maintenance of clinically relevant anxiety, it is of paramount importance to identify the involved cerebral structures for translational research.

On the one hand, the present study defines clear cut regions of interest for further studies on the cerebral mechanisms underlying neuropsychological attention trainings which both modify such cognitive biases and alleviate the symptoms in anxiety disorders [49]. In a similar way, the present findings may also bear relevance for future research on the neural underpinnings of cognitive behavioral therapy (CBT), the psychological gold standard therapy for anxiety disorders [50], as CBT reduces the attentional bias in anxiety disorders [51]. As the self-efficacy of cognitive reappraisal has been identified as mediator of CBT effects in SAD [52], studies investigating the effects of cognitive reappraisal on cognitive biases and their neural mediators in SA appear warranted to expand the scientific groundwork for further studies on the neural mediators of effective CBT for SAD.

Also, recently, it was demonstrated that a non-invasive electrophysiological intervention (i.e., anodal transcranial direct current stimulation (tDCS) applied to the left DLPFC impacts on processing of emotional cues in healthy subjects [53], [54] and, more importantly, reduces the attentional bias for emotional information in depression [55]. Moreover, a recent meta-analysis indicated a positive effect of left DLPFC tDCS on symptom reduction in depression [56]. Thus, the results of the present study represents a promising starting point for further research using laughter perception as an experimental probe to clarify the causal relationships between SA symptoms, attentional biases and the therapeutic effects of attention trainings, CBT and potentially tDCS.

Finally, Hofmann et al. [57] suggested modifying the activity of limbic and prefrontal brain areas using neurofeedback techniques in order to reduce anxious symptoms. In this context, the prefrontal brain regions identified in the present study represent a spatially well-defined target for the modification of both attention bias and SA via neurofeedback.

Although this study focused on SA, the localization of cerebral structures mediating cognitive biases may also be relevant for research on other psychiatric disorders as it has been shown that cognitive biases occur also in other anxiety disorders as well as depression [58], schizophrenia [59] and addiction [60].

Another outcome of the present study is the validation of a cognitive bias in SA during perception of nonverbal social signals using dynamic and multimodal cues. At the behavioral level, the lack of significant differences in the association of SA and the cognitive biases for different modalities of cue presentation fits with the observation of such biases both in the nonverbal [5]–[8] and verbal [61] domain of information processing and further supports the hypothesis that cognitive biases in SA solely depend on their relation to social threat and generalize across sensory modalities. Broadening the perspective to the neural mediators of aforesaid cognitive biases, differences between the cue presentation modalities become apparent: Our results demonstrate that the mediating effect left DLPFC activation is most prominently driven by patterns of activity occurring under bimodal (i.e., audiovisual) stimulation.

It is conceivable that this is due to the redundancy of social information from the auditory and visual channels in the bimodal cues which might help to shape more distinct cerebral activation patterns associated with this social information in supramodal frontal brain regions.

Apart from the fact that this stimulation condition approximates most closely natural social communication conditions, this finding additionally advocates the use of dynamic multimodal stimuli in social communication research.

Somewhat surprisingly, there was no significant association of the interpretation bias and cerebral activation patterns. From a conceptual point of view, it seems unlikely that the general interpretation bias during laughter perception should not have any neuronal correlate. There may, however, be a methodological reason for this negative result: Due to the lack of differences in the interpretation bias across the experimental conditions (i.e. laughter types and cue modalities), the analysis of its neuronal correlates was based on the fMRI main effect of laughter perception. This main effect, however, is contrasted against an implicit baseline and may therefore contain a greater amount of error variance than differential activations between laughter types, thus rendering the respective statistical analysis less sensitive. Here, the introduction of a suitable experimental baseline condition (e.g., filtered and unrecognizable laughter stimuli with preserved basic visual and auditory features) could represent a solution for this problem in future studies. Finally, while only a design with explicit social evaluation of the stimulus material allows the simultaneous rating-based and response time-based assessments of the relationship of SA and interpretation as well as attention biases, it may be useful in future studies to employ experimental designs with implicit (i.e., task-irrelevant) processing of social stimuli as these are known to elicit even more robust attention biases in SA.

## Limitations

Although mediation analysis affords a stronger basis for model-based causal inferences than “simple” correlational statistical approaches, it is nevertheless based on regression analysis and not completely free from the limitations bound to this type of analysis. Therefore, the verification of the causal nature of the observed activation patterns of DLPFC in future studies employing non-invasive electrophysiological and behavioral neuromodulatory techniques to actively modify cognitive biases in combination with fMRI appears as a logical and necessary extension of the present study.

A second limitation for the interpretability of our results can be found in the characteristics of the participant population. Although social anxiety typically has an early onset in life, the age distribution of the present sample as well as its high educational level somewhat limit the generalizability of our findings with respect to these parameters. Finally, the exclusion of psychiatric disorders other than SAD prevents the generalizability of the present results regarding individuals with comorbid forms of SAD.

## Conclusion

In summary, the novel application of laughter as a symptom provoking stimulus in SA and mediation analysis of fMRI data in the present study strongly supports the notion that altered functioning of the dorsal attention system represents the cerebral mediator of the negative attention bias in SA. Therefore, the present study can serve as a very useful basis for future investigations employing psychotherapeutic, neuropsychological, or electrophysiological interventions with influence on cognitive biases to clarify the causal relationships between psychiatric conditions, cognitive biases and altered cerebral functioning possibly even beyond the domain of anxiety disorders.

## Supporting Information

Table S1 **Brain areas where mean laughter ratings were negatively associated with mean cerebral responses during laughter perception.**
(DOC)

Table S2 **Exclusive significant mediation effect of cerebral activation patterns in the left DLPFC for the impact of social anxiety on the attentional bias during laughter perception (response times JOY – TAU) under bimodal (AV) stimulation in contrast to monomodal stimulation (A, V).**
(DOC)
