# Supplementary material for: They Are Laughing at Me: Cerebral Mediation of Cognitive Biases in Social Anxiety
Source: PLoS One. 2014 Jun 11;9(6):e99815. doi: 10.1371/journal.pone.0099815 (PMC4053467; doi:10.1371/journal.pone.0099815)
Supplement: Table S1 — Brain areas where mean laughter ratings were negatively associated with mean cerebral responses during laughter perception. (DOC) [file pone.0099815.s001.doc]

**Table S1** Brain areas where mean laughter ratings were negatively associated with mean cerebral responses during laughter perception.

|  | x | y | z | Z-score (peak voxel) | Cluster size (voxel) |
| --- | --- | --- | --- | --- | --- |
| L and R cuneus | 0 | -87 | 36 | 4.39 | 37 |
| R cerebellum/ R fusiform gyrus | -9 | -42 | 0 | 3.68 | 10 |
| L lingual gyrus/ L cerebellum | 27 | -60 | -18 | 3.52 | 10 |

Activations thresholded at p < 0.001, uncorrected with a cluster size k ≥ 10 voxels. Coordinates refer to the MNI system. None of the activations reached statistical significance at p < 0.05, FWE corrected for multiple comparisons across the whole brain at the cluster level corresponding to a cluster size of k > 77 voxels. Voxel size 3 x 3 x 3 mm³
