# Supplementary material for: They Are Laughing at Me: Cerebral Mediation of Cognitive Biases in Social Anxiety
Source: PLoS One. 2014 Jun 11;9(6):e99815. doi: 10.1371/journal.pone.0099815 (PMC4053467; doi:10.1371/journal.pone.0099815)
Supplement: Table S2 — Exclusive significant mediation effect of cerebral activation patterns in the left DLPFC for the impact of social anxiety on the attentional bias during laughter perception (response times JOY – TAU) under bimodal (AV) stimulation in contrast to monomodal stimulation (A, V). (DOC) [file pone.0099815.s002.doc]

**Table S2.** The mediation effect of cerebral activation (M) patterns in the left DLPFC for the impact of social anxiety (X) on the attentional bias (Y) during laughter perception (response times JOY – TAU) proved to be only significant for bimodal (AV) stimulation in contrast to monomodal stimulation (A, V).

| modality | control variable |  |  | X 🡪 Y |  | X 🡪 M | M 🡪 Y |
| --- | --- | --- | --- | --- | --- | --- | --- |
|  |  |  | total effect | direct effect | indirect effect |  |  |
|  |  |  | c | c’ | ab | a | b |
|  |  | beta | **4.142** | 2.048 | **2.094** | **0.0250** | **83.9** |
|  | none | Z | **3.2** | 1.8 | **2.4** | **2.7** | **3.2** |
|  |  | P value | **0.001** | 0.06 | **0.017** | **0.008** | **<0.001** |
|  |  | 95% CI |  |  | **4.216 – 0.479** |  |  |
|  |  | beta | **4.114** | 1.082 | **3.031** | **0.033** | **90.7** |
| AV | STAI-X1 | Z | **2.6** | 0.8 | **-** | **2.8** | **3.4** |
|  |  | P value | **0.010*** | 0.437 | **-** | **0.005** | **<0.001** |
|  |  | 95% CI |  |  | **5.909 – 0.961** |  |  |
|  |  | beta | **3.503** | 1.689 | **1.815** | **0.023** | **80.7** |
|  | STAI-X2 | Z | **2.6** | 1.5 |  | **2.2** | **3.1** |
|  |  | P value | **0.002** | 0.142 |  | **0.028** | **0.002** |
|  |  | 95% CI |  |  | **4.437 – 0.004** |  |  |
|  |  | beta | **2.315** | **1.979** | 0.336 | 0.005 | **72.5** |
|  | none | Z | **2.2** | **2.1** | 0.6 | 0.6 | **2.6** |
|  |  | P value | **0.031** | **0.034** | 0.534 | 0.541 | **0.008** |
|  |  | 95% CI |  |  | 2.019 – -0.913 |  |  |
|  |  | beta | 1.413 | 0.703 | 0.709 | 0.009 | **79.6** |
| A | STAI-X1 | Z | 1.1 | 0.7 |  | 0.9 | **3.0** |
|  |  | P value | 0.277 | 0.513 |  | 0.362 | **0.003** |
|  |  | 95% CI |  |  | 2.994 – -1.275 |  |  |
|  |  | beta | 1.668 | 1.591 | 0.077 | 0.001 | **67.1** |
|  | STAI-X2 | Z | 1.5 | 1.6 |  | 0.1 | **2.4** |
|  |  | P value | 0.139 | 0.112 |  | 0.890 | **0.016** |
|  |  | 95% CI |  |  | 1.499 – -1.344 |  |  |
|  |  | beta | 1.497 | 1.002 | 0.496 | 0.012 | 41.4 |
|  | none | Z | 1.1 | 0.7 | 1.0 | 1.6 | 1.0 |
|  |  | P value | 0.253 | 0.465 | 0.339 | 0.112 | 0.281 |
|  |  | 95% CI |  |  | 3.000 – -0.487 |  |  |
|  |  | beta | 1.245 | 0.776 | 0.470 | 0.011 | 41.2 |
| V | STAI-X1 | Z | 0.7 | 0.4 |  | 1.2 | 1.0 |
|  |  | P value | 0.460 | 0.654 |  | 0.239 | 0.295 |
|  |  | 95% CI |  |  | 3.605 – -0.504 |  |  |
|  |  | beta | 1.202 | 0.882 | 0.320 | 0.008 | 39.2 |
|  | STAI-X2 | Z | 0.8 | 0.6 |  | 0.7 | 1.0 |
|  |  | P value | 0.411 | 0.555 |  | 0.315 | 0.332 |
|  |  | 95% CI |  |  | 1.981 – -0.453 |  |  |

Mediation analysis: X = independent variable, Y = dependent variable, M = mediator, a = influence of X on M, b = influence of M on Y controlling for X, c = total effect of X on Y, c’ = direct effect of X on Y, ab = indirect effect of X on Y through M. Z values for the ab path pertain to the Sobel test while 95% CI pertain to the bootstrapping approach (for details see Methods). Values in bold print refer to significant effects.
